# Predictors of short-term successful discontinuation of continuous renal replacement therapy: results from a prospective multicentre study

**DOI:** 10.1186/s12882-019-1327-9

**Published:** 2019-04-15

**Authors:** Susanne Stads, K. Merijn Kant, Margriet F. C. de Jong, Wouter de Ruijter, Christa M. Cobbaert, Michiel G. H. Betjes, Diederik Gommers, Heleen M. Oudemans-van Straaten

**Affiliations:** 1 Department of Intensive Care, Erasmus Medical Centre, Rotterdam, Netherlands; 2 Department of Intensive Care, Ikazia Hospital, Rotterdam, Netherlands; 3 Department of Intensive Care, Amphia Hospital, Breda, Netherlands; 4 Department of Nephrology, University Medical Centre Groningen, Groningen, Netherlands; 5 Department of Intensive Care, Noordwest ziekenhuisgroep Alkmaar, Alkmaar, Netherlands; 6 Department of Clinical Chemistry and Laboratory Medicine, Leiden University Medical Centre, Leiden, Netherlands; 7 Department of Nephrology, Erasmus Medical Centre, Rotterdam, Netherlands; 8 Department of Intensive Care, Amsterdam UMC, Vrije Universiteit Amsterdam, Amsterdam, Netherlands

**Keywords:** Acute kidney injury, Discontinuation, Continuous renal replacement therapy, Neutrophil gelatinase-associated Lipocalin, Prediction

## Abstract

**Background:**

Prediction of successful discontinuation of continuous renal replacement therapy (CRRT) might reduce complications of over- and under-treatment. The aim of this study was to identify renal and non-renal predictors of short-term successful discontinuation of CRRT in patients in whom CRRT was stopped because renal recovery was expected and who were still in the Intensive Care Unit (ICU) at day 2 after stop CRRT.

**Methods:**

Prospective multicentre observational study in 92 patients alive after discontinuation of CRRT for acute kidney injury (AKI), still in the ICU and free from renal replacement therapy (RRT) at day 2 after discontinuation. Successful discontinuation was defined as alive and free from RRT at day 7 after stop CRRT. Urinary neutrophil gelatinase-associated lipocalin (NGAL) and clinical variables were collected. Logistic regression and Receiver Operator Characteristic (ROC) curve analysis were performed to determine the best predictive and discriminative variables.

**Results:**

Discontinuation of CRRT was successful in 61/92 patients (66%). Patients with successful discontinuation of CRRT had higher day 2 urine output, better renal function indicated by higher creatinine clearance (6-h) or lower creatinine ratio (day 2/day 0), less often vasopressors, lower urinary NGAL, shorter duration of CRRT and lower cumulative fluid balance (day 0–2). In multivariate analysis renal function determined by creatinine clearance (Odds Ratio (OR) 1.066, 95% confidence interval (CI) 1.022–1.111, *p* = 0.003) or by creatinine ratio (day 2/day 0) (OR 0.149, 95% CI 0.037–0.583, *p* = 0.006) and non-renal sequential organ failure assessment (SOFA) score (OR 0.822, 95% CI 0.678–0.996, *p* = 0.045) were independently associated with successful discontinuation of CRRT. The area under the curve of creatinine clearance to predict successful discontinuation was 0.791, optimal cut-off of 11 ml/min (95% CI 6–16 ml/min) and of creatinine ratio 0.819 (95% CI 0.732–0.907) optimal cut-off of 1.41 (95% CI 1.27–1.59).

**Conclusion:**

In this prospective multicentre study we found higher creatinine clearance or lower creatinine ratio as best predictors of short-term successful discontinuation of CRRT, with a creatinine ratio of 1.41 (95% CI 1.27–1.59) as optimal cut-off. This study provides a practical bedside tool for clinical decision making.

## Background

Acute kidney injury (AKI) is a common complication of critical illness and patients requiring renal replacement therapy have excess mortality even when adjusted for severity of disease [1–4]. The optimal timing to start continuous renal replacement therapy (CRRT) has been investigated in several studies. The urinary biomarker Neutrophil gelatinase-associated lipocalin (NGAL) has high potential as an early predictor of severe AKI [5, 6]. However, only few studies are available on the use of biomarkers to predict successful discontinuation of CRRT [7–9].

In daily practice, CRRT is discontinued on an individual basis: when urinary output increases or when the CRRT session ends and the attending physician supposes that renal function will recover because other organ functions improve. Previous studies found that lower age, less severe organ failure, shorter duration of CRRT, higher creatinine clearance or urine output during CRRT and decreasing plasma NGAL on the first day of RIFLE-F were associated with recovery [4, 7–15]. Clinical reasons for re-initiation of CRRT are fluid overload, hyperkalaemia and azotaemia [15]. However, none of these studies evaluated biomarkers at discontinuation of CRRT.

Predicting short-term successful discontinuation in patients in whom CRRT has been stopped may prevent potentially harmful complications of over- and under treatment. We hypothesized that high urine output, high endogenous creatinine clearance or low creatinine ratio, low urinary NGAL, no vasopressor use and low non-renal sequential organ failure assessment (SOFA) score after discontinuation are associated with successful discontinuation of CRRT. The objectives of the present study were to identify renal and non-renal predictors for short-term successful discontinuation.

## Methods

### Study design

We performed a prospective multicentre observational study in 4 intensive care units (ICUs) in the Netherlands (Erasmus (University) Medical Centre, Rotterdam, Ikazia Hospital Rotterdam, Amphia Hospital Breda and Medical Centre Alkmaar). Patients were included from May 2013 until September 2015. The protocol was approved by the medical ethics committee of the Erasmus Medical Centre and the local ethical committees. Written informed consent was obtained from all participants or their legal representative.

### Patients

All patients aged 18 years or older, alive and still admitted to the ICU at day 2 after discontinuation of CRRT were screened for eligibility. Patients with end-stage-renal-disease (CKD 5) with or without chronic renal replacement therapy, and patients receiving CRRT for other reasons than acute renal failure (e.g. liver failure, intoxications) were excluded. Patients discharged from the ICU before day 2 were excluded from analysis, because primary study variables could not be collected from these patients.

### Sample size calculation

For evaluation of predictors of short-term successful discontinuation, we defined five primary study variables (urine output, renal function determined by calculated creatinine clearance or creatinine ratio, urinary NGAL, vasopressor use and non-renal SOFA score) which were hypothesized predictive and two secondary study variables (duration of CRRT and cumulative fluid balance) which were derived from the literature. We planned to test a total of 7 variables in multivariate regression analysis and therefore aimed to include at least 70 evaluable patients as suggested by Altman (“no more than n/10 variables, where n is the sample size” [16]). Because of expected exclusions caused by early discharge and missing urine samples we aimed to include 90 patients.

### Study protocol and measurements

**Fig. 1:**
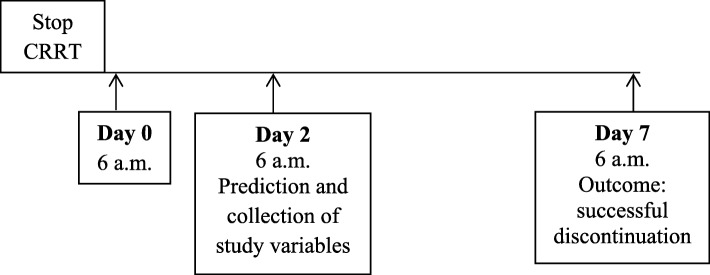
study outline

Successful discontinuation was defined as alive and free from RRT at day 7 after discontinuation. We chose 2 days after discontinuation of CRRT as time point to predict whether discontinuation of CRRT would be successful for the subsequent 5 days to include only those patients for whom the prediction of successful discontinuation has direct logistical consequences for the unit, and to evaluate only the patients in whom CRRT was discontinued because of expected renal recovery and not those in whom CRRT was temporarily discontinued for logistical reasons (such as CT scan or surgery) or switch of dialysis modality to intermittent haemodialysis. Day 0 was defined as the first 6 a.m. after discontinuation of CRRT. Day 7 as the day at which the outcome successful discontinuation was determined (Fig. 1). The decision to (re)initiate or discontinue renal replacement therapy in the ICU was made according to the decision of the local team. CRRT was performed according to the local protocol of the hospital as continuous venovenous hemofiltration (CVVH) or continuous venovenous haemodialysis (CVVHD) and delivered dose was 20–35 ml/kg/hour. We used polyethersulfone, acrylonitrile/ sodium methallyl sulfonate polymer membranes with a surface area of 1.8 m^2^–1.9 m^2^ and an in vitro cut-off point of 30–55 kDa, depending on local availability of materials.

### Study variables

The following *primary study variables* were collected at day 2: urine output, renal function determined by 6-h endogenous creatinine clearance and incremental creatinine ratio, urinary NGAL concentration (when diuresis was > 200 ml/day) normalized to urinary creatinine concentration, vasopressor use and non-renal SOFA score. Renal function was determined by calculation of creatinine clearance according to the following formula: ((urinary creatinine concentration * urine volume)/ plasma creatinine concentration)/ 360, and calculation of the incremental creatinine ratio between day 2 and day 0 (at discontinuation) (creatinine day 2/day 0). The following *secondary study variables* were collected on day 2 as well: duration of CRRT (as found in previous studies [10, 11, 14, 15]) and cumulative fluid balance from day 0 until day 2 (as used in clinical practice as reason for restart).

### Other measurements

The following variables were determined at start of CRRT: demographic data, preadmission creatinine (defined as creatinine 1 month prior to admission or more without disease), preadmission estimated glomerular filtration rate (eGFR) (calculated with CKD-EPI formula [17]), previous kidney disease, reason for ICU admission (post-operative, respiratory failure, sepsis, post cardiac arrest, neurologic, cardiac failure), disease severity scores (Acute Physiology And Chronic Health Evaluation (APACHE) III, Simplified Acute Physiology Score (SAPS) III), cause of AKI (defined as sepsis, toxic, primary renal disease, ischemic/other).

### Endpoints

The primary endpoint was successful discontinuation, defined as alive and free from any form of RRT at day 7 after stop CRRT.

### Assays

For determination of NGAL, a tube collected from a 6 h urine portion was stored in the refrigerator for a maximum of 72 h. As soon as possible the sample was centrifuged for 10 min at 2000G at 4 °C and the supernatant was stored at − 80 °C for determination of urinary NGAL later. Urinary NGAL was determined by immunoassay using the Architect ci4100 (Abbott Diagnostics, Abbott Park, IL, US), we used the Urine NGAL Rgt 100 T (1P37–25), NGAL Calibrator (1P37–01), NGAL Controle (1P37–10) according to manufacturer’s specifications. NGAL values were normalized to creatinine concentration and expressed as (ng/ml)/creatinine (mmol/L).

### Statistical analysis

Variables were tested for normal distribution using the Kolmogorov-Smirnov test. Normally distributed variables are expressed as mean (standard deviation), non-normally distributed variables as median [25th and 75th percentile], and categorical data as number and percentage. Unpaired Student’s t-test, Mann-Whitney-U test or Chi-square test was used, where appropriate. Statistical significance was defined as *p* < 0.05.

To determine the association between the primary study variables (urine output, endogenous creatinine clearance, urinary NGAL, use of vasopressors and non-renal SOFA score) and the secondary study variables (duration of CRRT and fluid balance day 0–2) with successful discontinuation of CRRT, univariate logistic regression analysis was performed and subsequent multivariate analysis was performed. For all analyses, multicollinearity was checked with a maximum variance inflation factor (VIF) of 10.

A ROC curve was drawn for the best discriminative variable of successful discontinuation of CRRT. The area under the receiver operator characteristic curve (AUC) was calculated to discriminate for successful discontinuation of CRRT. The Youden index was calculated to determine the optimal cut-off to discriminate for successful discontinuation. The confidence interval for the optimal cut-off was calculated using bootstrapping with 1000 random samples using the bias corrected and accelerated method.

## Results

### Flowchart

**Fig. 2:**
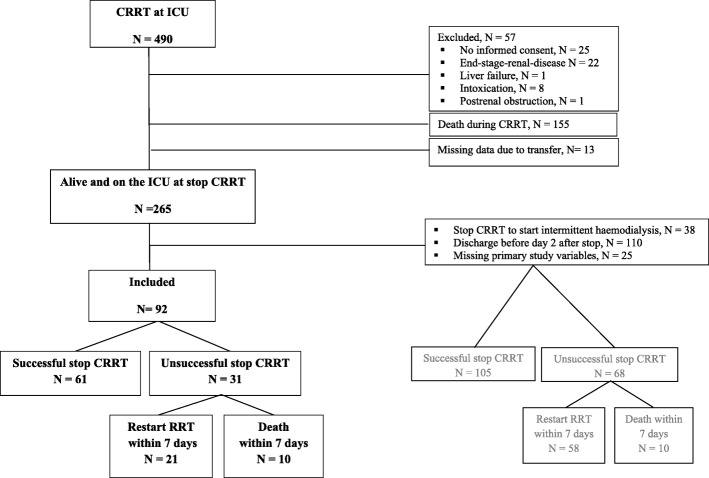
flowchart of included and excluded patients

Of the 490 patients receiving CRRT during the study period, 57 patients met the exclusion criteria, 155 patients died during CRRT, 13 patients were transferred to another hospital during CRRT, 110 patients were discharged from the ICU before the predefined sampling point at day 2, 25 patients had missing primary study variables and in 38 patients CRRT was discontinued because of switch of modality to intermittent haemodialysis. Among the 92 included patients, 61 patients (66%) experienced successful discontinuation of CRRT at day 7, 21 patients (23%) needed re-initiation of RRT before day 7, and 10 patients (11%) died within 7 days after discontinuation of CRRT (Fig. 2). Of the 173 excluded patients who were alive and on the ICU at discontinuation of CRRT, 105 patients (61% of excluded patients) experienced successful discontinuation, 58 patients (33% of excluded patients) needed re-initiation of RRT and 10 patients (6% of excluded patients) died within 7 days (Fig. 2). In 38/58 patients needing restart of RRT, CRRT was discontinued for switch to intermittent haemodialysis.

Differences between included and excluded patients are depicted in Table 1. Excluded patients had worse preadmission renal function compared to included patients. Furthermore re-initiation of intermittent haemodialysis was needed in 38 patients (22%) of the excluded patients compared to 4 patients (4%) of the included patients.

**Table 1:** Baseline characteristics for included and excluded patients

	Included patients *n* = 92	Excluded patients *n* = 173	*P*-value
Age (years)	66 [55, 74]	64 [57, 74]	0.620
Male gender, nr (%)	55 (60)	126 (73)	0.030
Weight (kg)	82 (16)	80 [71, 95]	0.693
BMI (kg/m^2^)	25.7 [23.5, 30.3]	27.0 [23.7, 30.9]	0.522
Previous kidney disease, nr (%)	20 (22)	57 (32)	0.056
Preadmission creatinine, μmol/L	96 [74, 127]	103 [83, 149]	0.030
Preadmission eGFR (CKD-EPI), (ml/min/1.73m^2^)	67.8 (26.2)	60.8 (26.7)	0.047
Cause of AKI, nr (%)			
Sepsis	34 (37)	51 (30)	0.052
Toxic	4 (4)	24 (14)	
Primary renal disease	2 (2)	8 (5)	
Ischemic/ other	52 (57)	90 (52)	
Reason for ICU admission, nr (%)			
Post-operative	32 (35)	53 (31)	0.470
Respiratory failure	15 (16)	24 (14)	
Sepsis	17 (19)	30 (17)	
Post cardiac arrest	7 (7)	14 (8)	
Cardiac failure	9 (10)	20 (12)	
Other	12 (13)	32 (19)	
SAPS III admission	53 (16)	54 (15)	0.313
APACHE III admission	89 (31)	81 [66, 103]	0.188
Mechanical ventilation at initiation of CRRT	69 (75)	109 (63)	0.107
Vasopressor at initiation of CRRT	74 (80)	117 (68)	0.041
Successful stop CRRT, nr (%)			
Yes	60 (65)	105 (61)	0.001
No, restart of CRRT	18 (20)	20 (11)	
No, restart of IHD	4 (4)	38 (22)	
No, death	10 (11)	10 (6)	

Mean (standard deviation) for normally distributed variables, median [25th and 75th percentile] for non-normally distributed variables, number (percentage) when appropriate

*BMI* body mass index, *eGFR* estimated glomerular filtration rate, *CKD-EPI* chronic kidney disease epidemiology collaboration, *AKI* acute kidney injury, *ICU* intensive care unit, *SAPS III* simplified acute physiology score, *APACHE III* acute physiology and chronic health evaluation score, *CRRT* continuous renal replacement therapy, *IHD* intermittent haemodialysis

### Patient characteristics according to successful or unsuccessful discontinuation

Demographic and baseline characteristics at CRRT initiation were not significantly different between patients with successful and unsuccessful discontinuation of CRRT (Table 2). Accordingly, events during and after CRRT, such as new infections, the use of nephrotoxic medication or intravenous contrast, use of diuretics and the use of citrate anticoagulation were not significantly different between patients with successful and unsuccessful discontinuation of CRRT (Table 2).

**Table 2:** Demographic and baseline characteristics at start of CRRT and events during or after CRRT according to successful or unsuccessful discontinuation

	All patients *n* = 92	Successful stop CRRT *n* = 61 (66%)	Unsuccessful stop CRRT *n* = 31 (34%)	*P*-value
Age (years)	66 [55, 74]	62 (13)	64 (14)	0.275
Male gender, nr (%)	55 (60)	38 (62)	17 (55)	0.491
Weight (kg)	82 (16)	84 (17)	79 (15)	0.153
BMI (kg/m^2^)	25.7 [23.5, 30.3]	26.5 [23.9, 32.1]	25.1 [22.9, 27.7]	0.146
Previous kidney disease, nr (%)	20 (22)	10 (16)	10 (32)	0.081
Preadmission creatinine, μmol/L	96 [74, 127]	101 (39)	96 [71, 139]	0.599
Preadmission eGFR (CKD-EPI), (ml/min/1.73m^2^)	67.8 (26.2)	69.5 (25.1)	64.1 (46.0)	0.375
Cause of AKI, nr (%)				
Sepsis	34 (37)	19 (31)	15 (48)	0.058
Toxic	4 (4)	3 (5)	1 (3)	
Primary renal disease	2 (2)	0 (0)	2 (7)	
Ischemic/ other	52 (57)	39 (64)	13 (42)	
Reason for ICU admission, nr (%)				
Post-operative	32 (35)	20 (33)	12 (39)	0.185
Respiratory failure	15 (16)	10 (17)	5 (16)	
Sepsis	17 (19)	8 (13)	9 (29)	
Post cardiac arrest	7 (7)	7 (11)	0 (0)	
Cardiac failure	9 (10)	7 (11)	2 (6)	
Other	12 (13)	9 (15)	3 (10)	
SAPS III admission	53 (16)	52 (15)	55 (18)	0.470
APACHE III admission	89 (31)	88 (29)	91 (36)	0.624
Mechanical ventilation at initiation of CRRT	69 (75)	45 (74)	24 (77)	0.702
Vasopressor at initiation of CRRT	74 (80)	49 (80)	25 (81)	0.971
Events during or after CRRT				
Infection day −4 until day 0, nr (%)	21 (23)	14 (24)	7 (23)	0.936
Infection day 0 until day 7 after stop CRRT, nr (%)	17 (19)	9 (15)	8 (26)	0.197
Citrate anticoagulation, nr (%)	74 (80)	49 (80)	25 (81)	0.971
Nephrotoxic medication or IV contrast, day −4 until day 0, nr (%)	68 (75)	43 (71)	25 (81)	0.350
Nephrotoxic medication or IV contrast, day 0 until day 7 after stop, nr (%)	62 (67)	43 (71)	19 (61)	0.374
Diuretics day 0 until day 2, nr (%)	73 (80)	45 (74)	28 (90)	0.064

Mean (standard deviation) for normally distributed variables, median [25th and 75th percentile] for non-normally distributed variables, number (percentage) when appropriate

*BMI* body mass index, *eGFR* estimated glomerular filtration rate, *CKD-EPI* chronic kidney disease epidemiology collaboration, *AKI* acute kidney injury, *ICU* intensive care unit, *SAPS III* simplified acute physiology score, *APACHE III* acute physiology and chronic health evaluation score, *CRRT* continuous renal replacement therapy

At day 2 after stop CRRT, patients with successful discontinuation of CRRT had higher urine output, 2.424 (1.232) L vs. 1.640 (1.217) L (*p* = 0.005), higher creatinine clearance, 29 [14, 56] ml/min vs. 7 [4, 16] ml/min (*p* < 0.001), lower creatinine ratio (day 2 / day 0), 1.16 [0.91, 1.39] vs. 1.63 [1.42, 1.82] (*p* < 0.001), lower urinary NGAL(ng/ml)/creatinine (mmol/L), 80 [10, 249] vs. 583 [203, 1027] (ng/ml)/ creatinine (mmol/L) (*p* = 0.002), less often vasopressors 10 (16%) vs. 12 (39%) (*p* = 0.018) compared to patients with unsuccessful discontinuation of CRRT. Furthermore, patients with successful discontinuation of CRRT had shorter duration of CRRT, 4 [3, 9] days vs. 7 [4, 17] days (*p* = 0.014), and negative cumulative fluid balance between day 0 and 2, − 1.284 (2.884) L vs. 1.250 (2.942) L (*p* < 0.001) (Table 3).

**Table 3:** Potential predictors of successful discontinuation collected on day 2

	All patients *n* = 92	Successful stop CRRT *n* = 61 (66%)	Unsuccessful stop CRRT *n* = 31 (34%)	*P*-value
Primary study variables				
Urine output (L)	2.160 (1.276)	2.424 (1.232)	1.640 (1.217)	0.005
Creatinine clearance, ml/min	20 [7, 41]	29 [14, 56]	7 [4, 16]	< 0.001
Creatinine ratio (day 2/ day 0)	1.35 [1.06, 1.63]	1.16 [0.91, 1.39]	1.63 [1.42, 1.81]	< 0.001
Urinary NGAL (ng/ml)/ creatinine (mmol/L) (n = 63)	152 [15, 601]	80 [10, 249]	583 [203, 1027]	0.002
Vasopressor use, nr (%)	22 (24)	10 (16)	12 (39)	0.018
Non-renal SOFA score	4 [3, 7]	4 [3, 5]	6 (3)	0.080
Secondary study variables				
Duration of CRRT (days)	5 [3, 10]	4 [3, 9]	7 [4, 17]	0.014
Cumulative fluid balance, day 0–2 (L)	−0.430 (3.129)	−1.284 (2.884)	1.250 (2.942)	< 0.001

Mean (standard deviation) for normally distributed variables, median [25th and 75th percentile] for non-normally distributed variables, number (percentage) when appropriate

*NGAL* neutrophil gelatinase-associated lipocalin, *SOFA* sequential organ failure assessment, *CRRT* continuous renal replacement therapy

### Association between day 2 variables and successful discontinuation of CRRT

In univariate regression analysis we found a significant association between successful discontinuation of CRRT and higher day 2 urine output (OR 1.777, 95% CI 1.168–2.704, *p* = 0.007), higher creatinine clearance (OR 1.069, 95% CI 1.030–1.109, *p* < 0.001), lower incremental creatinine ratio (day 2/ day 0) (OR 0.100, 95% CI 0.027–0.370, *p* = 0.001), lower urinary NGAL (ng/ml)/creatinine (mmol/L) (OR 0.998, 95% CI 0.997–1.000, *p* = 0.025), no vasopressor use (OR 0.310, 95% CI 0.115–0.836, *p* = 0.021), shorter CRRT duration (OR 0.926, 95% CI 0.868–0.987, *p* = 0.018) and lower cumulative fluid balance (OR 0.734, 95% CI 0.618–0.876, *p* = 0.001). Non-renal SOFA score was associated with successful discontinuation although non-significantly (Table 4).

**Table 4:** Univariate analysis of variables associated with successful discontinuation (*n* = 92)

	OR	95% CI	*P*-value
Primary study variables			
Urine output (L) (day 2)	1.777	1.168–2.704	0.007
Creatinine clearance, ml/min (day 2)	1.069	1.030–1.109	< 0.001
Creatinine ratio (day 0 / day2)	0.100	0.027–0.370	0.001
Urinary NGAL (ng/ml)/creatinine (mmol/L) (day 2) (*n* = 63)	0.998	0.997–1.000	0.025
Vasopressor use (day 2)	0.310	0.115–0.836	0.021
Non-renal SOFA score (day 2)	0.854	0.729–1.001	0.052
Secondary study variables			
Duration of CRRT (days)	0.926	0.868–0.987	0.018
Cumulative fluid balance, day 0–2 (L)	0.734	0.618–0.876	0.001

*OR* Odds ratio; The odds ratios are per unit increase, *95% CI* 95% confidence interval, *NGAL* neutrophil gelatinase-associated lipocalin, *SOFA* sequential organ failure assessment, *CRRT* continuous renal replacement therapy

Urinary NGAL was only available in 63 patients. Reasons for missing values were anuria or failure to collect or store the urine portion. The primary multivariate analysis was performed in the entire group (n = 92), because inclusion of urinary NGAL would cause a substantial loss of data. The analysis showed that a higher day 2 calculated creatinine clearance (OR 1.066, 95% CI 1.022–1.111, *p* = 0.003), and a lower non-renal SOFA score (OR 0.822, 95% CI 0.678–0.996, *p* = 0.045) were significantly associated with successful discontinuation of CRRT, a negative fluid balance contributed non-significantly (OR 0.848, 95% CI 0.708–1.014, *p* = 0.071) (Table 5).

**Table 5:** Primary multivariate analysis of variables associated with successful discontinuation of CRRT (*n* = 92)

	OR	95% CI	*P*-value
Creatinine clearance, ml/min (day 2)	1.066	1.022–1.111	0.003
Non-renal SOFA (day 2)	0.822	0.678–0.996	0.045
Cumulative fluid balance, day 0–2 (L)	0.848	0.708–1.014	0.071

Variables included: Urine output (day 2), creatinine clearance (day 2), vasopressor use (day 2), non-renal SOFA score (day 2), duration of CRRT (days), cumulative fluid balance, day 0–2

Variables removed: step 2: Urine output (day 2) was lost, step 3: duration of CRRT was lost, step 4: Vasopressor use (day 2) was lost

Nagelkerke R^2^ of final model 0.415

*OR* Odds ratio; The odds ratios are per unit increase, *95% CI* 95% confidence interval, *SOFA* sequential organ failure assessment

A second multivariate analysis in the entire group (*n* = 92) including incremental creatinine ratio (day 2/ day 0) instead of creatinine clearance, showed that a lower creatinine ratio (day 2/ day 0) (OR 0.149, 95% CI 0.038–0.583, *p* = 0.006) and a lower non-renal SOFA score (OR 0.836, 95% CI 0.700–1.000, *p* = 0.049) were significantly associated with successful discontinuation of CRRT, a negative fluid balance contributed non-significantly (OR 0.830, 95% CI 0.686–1.005, *p* = 0. 057) (Table 6). The Nagelkerke R^2^ of the latter model was lower than that of the model including creatinine clearance. There was no significant collinearity, the VIF were < 3 for all combinations of variables.

**Table 6:** Second multivariate analysis of variables associated with successful discontinuation of CRRT (*n* = 92)

	OR	95% CI	*P*-value
Creatinine ratio (day 2 / day 0)	0.149	0.038–0.583	0.006
Non-renal SOFA (day 2)	0.836	0.700–1.000	0.049
Cumulative fluid balance, day 0–2 (L)	0.830	0.686–1.005	0.057

Variables included: Urine output (day 2), creatinine ratio (day 2 / day 0), vasopressor use (day 2), non-renal SOFA score (day 2), duration of CRRT (days), cumulative fluid balance, day 0–2

Variables removed: step 2: Vasopressor use (day 2) was lost, step 3: duration of CRRT was lost, step 4: Urine output (day 2) was lost

Nagelkerke R^2^ of final model 0.356

*OR* Odds ratio; The odds ratios are per unit increase, *95% CI* 95% confidence interval, *SOFA* sequential organ failure assessment

A sensitivity analysis was performed in the group in which urinary NGAL(ng/ml)/creatinine (mmol/L) was available (*n* = 63). In this analysis a higher creatinine clearance (OR 1.088, 95% CI 1.028–1.151), *p* = 0.004, and lower non-renal renal SOFA (OR 0.764, 95% CI 0.604–0.765), *p* = 0.024, were significant predictors of successful discontinuation. Urinary NGAL (ng/ml)/creatinine (mmol/L) was removed in step 3 (Table 7).

**Table 7:** Multivariate sensitivity analysis including only the patients with available urinary NGAL (63 patients)

	OR	95% CI	*P*-value
Creatinine clearance, ml/min (day 2)	1.088	1.028–1.151	0.004
Non-renal SOFA (day 2)	0.764	0.604–0.965	0.024

Variables included: Urine output (day 2), creatinine clearance, ml/min (day 2), Urinary NGAL (day 2) (ng/ml)/creatinine (mmol/L), vasopressor use (day 2), non-renal SOFA score (day 2), cumulative fluid balance day 0–2, duration of CRRT (days)

Variables removed: step 2: Urine output (day 2) was lost, step 3: Urinary NGAL (ng/ml)/creatinine (mmol/L) (day 2) was lost, step 4: Vasopressor use (day 2) was lost, step 5: duration of CRRT was lost, Step 6: cumulative fluid balance day 0–2 was lost

Nagelkerke R^2^ of final model (*n* = 63) 0.406

*OR* Odds ratio; The odds ratios are per unit increase, *SOFA* sequential organ failure assessment

### Discrimination of renal function determined as creatinine clearance or creatinine ratio (day 2/day 0) to predict successful discontinuation of CRRT

**Fig. 3:**
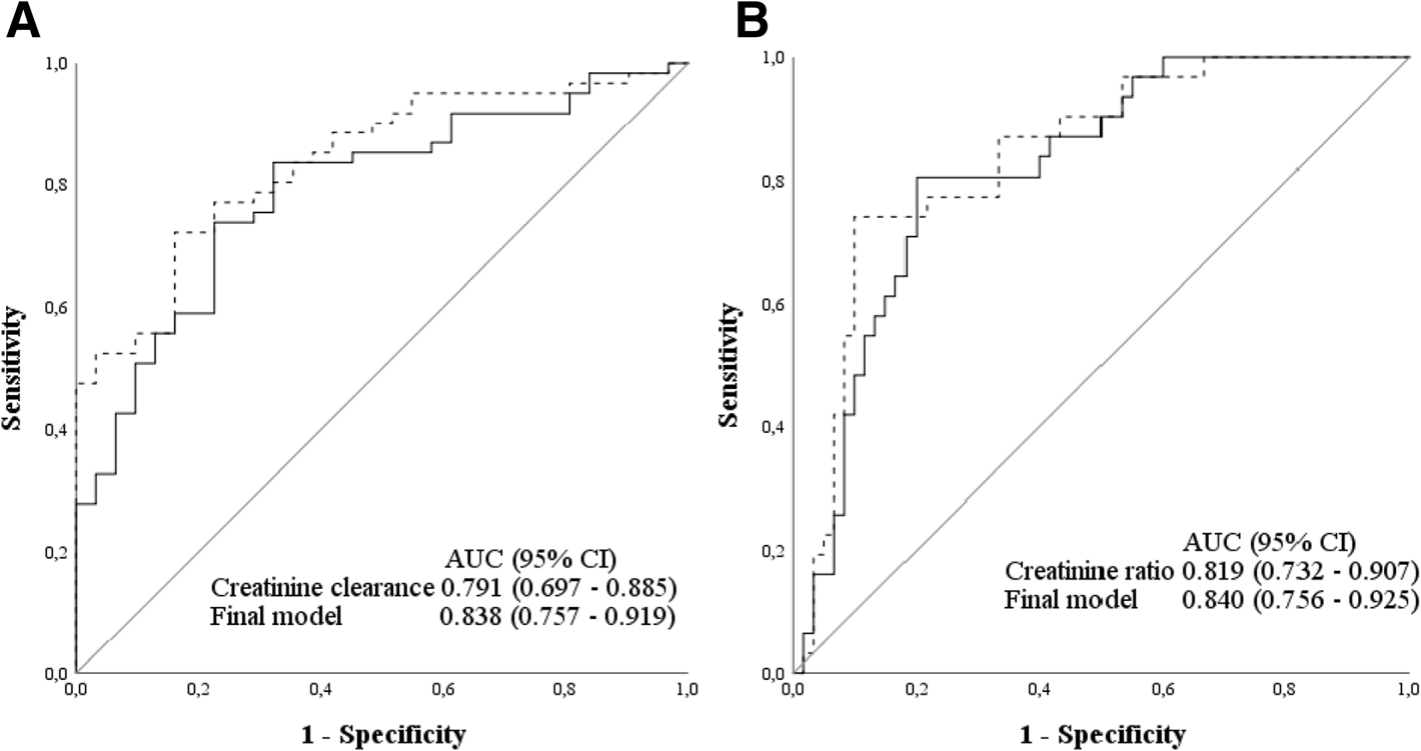
ROC curve for the prediction of successful discontinuation of CRRT. **a** ROC curve for creatinine clearance and final model. Creatinine clearance (black solid line) and the final model including creatinine clearance, non-renal SOFA score and cumulative fluid balance (dotted line). **b** ROC curve for creatinine ratio (day 2/day 0) and final model. Creatinine ratio (day 2/ day 0) (black solid line) and the final model including creatinine ratio (day 2/ day 0), non-renal SOFA score and cumulative fluid balance (dotted line)

The AUC of the ROC curve for creatinine clearance to discriminate for short-term successful discontinuation of CRRT was 0.791 (95% CI 0.697–0.885) (Fig. 3a), with an optimal cut-off of 11 ml/min (95% CI 6–16 ml/min), sensitivity 0.84, specificity 0.68, positive predictive value of 84% and negative predictive value of 68%. The AUC of the ROC curve of the final model, including creatinine clearance, non-renal SOFA score and cumulative fluid balance, was 0.838 (95% CI 0.757–0.919) (Fig. 3a).

The AUC of the ROC curve for creatinine ratio (day 2/day 0) to discriminate for short-term successful discontinuation of CRRT was 0.819 (95% CI 0.732–0.907) (Fig. 3b), with an optimal cut-off of 1.41 (95% CI 1.27–1.59), sensitivity 0.80, specificity 0.81, positive predictive value 89% and negative predictive value of 68%. The AUC of the ROC curve of the final model including creatinine ratio (day 2/ day 0), non-renal SOFA score and cumulative fluid balance is 0.840 (0.756–0.925) (Fig. 3b).

## Discussion

In this prospective multicentre study in critically ill patients receiving CRRT for AKI, we found that a better renal function determined by a higher day 2 creatinine clearance or a lower creatinine ratio (day 2/ day 0) and a lower non-renal SOFA score after discontinuation of CRRT were independently associated with successful discontinuation of CRRT at day 7. A less positive fluid balance improved the model, but the association was not significant. With an AUC of 0.791, the discrimination of creatinine clearance was good, with an optimal cut off for successful discontinuation of 11 ml/min (95% CI 6–16 ml/min), and with an AUC of 0.819, the discrimination of creatinine ratio (day 2/day 0) was also good, with an optimal cut-off for successful discontinuation of 1.41 (95% CI 1.27–1.59). On univariate analysis, urine output, urinary NGAL (ng/ml)/creatinine (mmol/L), duration of CRRT and vasopressor use were significantly associated with successful discontinuation, but these variables were removed in multivariate analysis. Of interest, in patients with successful discontinuation cumulative fluid balance (day 0–2) was negative, while in patients with unsuccessful discontinuation cumulative fluid balance (day 0–2) was positive, whereas the use of diuretics was not significantly different between patients exhibiting successful vs. unsuccessful discontinuation. The latter were apparently not able to remove fluid due to insufficient renal function, persistent associated organ failure or both.

Nowadays there are no guidelines on when CRRT in the ICU can be discontinued. Physicians decide on individual basis, based on bedside parameters or logistic factors, for example when the patient has to undergo a CT scan or when the circuit has to be replaced. In the present study, previously defined variables were prospectively collected from all included patients. The finding that calculated creatinine clearance from a 6-h portion was the best predictor is important because it is an easy and cheap marker and available within hours. When creatinine clearance is higher than 16 ml/min, the upper limit of the 95% confidence interval, successful discontinuation is likely. In case of doubt, when creatinine is within the 95% confidence limits, SOFA score and fluid balance can be considered. A higher SOFA score or a more positive fluid balance could be an argument for restart.

The creatinine ratio (day 2/ day 0) was also predictive, however, the multivariate model including creatinine clearance was more sensitive. Creatinine ratio is presently used to classify the severity of AKI according to the KDIGO guidelines during its development [18]. In these guidelines a creatinine ratio from 1.5–1.9 times baseline is defined as AKI stage 1. In the present study we found that an incremental creatinine ratio (day 2/day 0) below 1.41 discriminated for short-term successful discontinuation. Our study therefore provides practical and plausible tools for clinical decision making.

Up to now, only retrospective studies or a post hoc analysis evaluated the association between variables at discontinuation of CRRT and successful discontinuation. Urine output was the best predictor of successful discontinuation of CRRT in two studies evaluating current practice on discontinuation of CRRT [10, 11]. Only one study also evaluated the association between creatinine clearance and successful discontinuation of CRRT. This study used a 2-h creatinine clearance and found an optimal cut-off of 23 ml/min. However, this retrospective study evaluated calculated creatinine clearance in the 12 h preceding discontinuation and did not evaluate the contribution of SOFA score to the final model. Furthermore the reasons to re-initiate RRT might have been different [13]. We prospectively confirmed that measuring creatinine clearance is the best predictor of short-term successful discontinuation.

Urinary NGAL was lower in patients with successful discontinuation, suggesting less kidney damage. However, contrary to expectation the association between urinary NGAL and successful discontinuation was non-significant in multivariate analysis. Urinary NGAL is a promising early biomarker predicting AKI and need of RRT [5, 19, 20]. When determined 24 h after AKI diagnosis, a decline in plasma NGAL was associated with renal recovery after 48 h [7]. Interestingly, kinetic eGFR calculation after initial resuscitation discriminated better for renal recovery from AKI without RRT than urinary biomarkers NGAL or [TIMP-2]*[IGFBP7]. Creatinine clearance, creatinine ratio (day 2/ day 0) and kinetic eGFR reflect actual renal function while NGAL and [TIMP-2]*[IGFBP7] reflect renal damage and cell cycle arrest respectively. Thus a marker of renal function seems more predictive than a marker of renal damage.

In multivariate analysis non-renal SOFA score was associated with successful discontinuation of CRRT as well, suggesting that patients with less severe illness at discontinuation of CRRT are more likely to experience successful discontinuation of CRRT. A high severity of disease has been reported as being associated with non-recovery of renal function or re-initiation of CRRT before [12, 14]. Our study is the first to evaluate the role of fluid balance as a marker of successful discontinuation. Incorporating fluid balance in the model improved its prediction, although not significantly.

Our study has several limitations, first despite screening a large group of patients receiving CRRT only a small group was included in the final analysis, mainly because of mortality during or shortly after discontinuation of CRRT, and early discharge to the ward. Therefore we compared the excluded patients who were alive and on the ICU at discontinuation with the included patients and found that in a large group of the excluded patients (38/173), CRRT was discontinued for switch to intermittent haemodialysis and not because of expected recovery of renal function. Furthermore these excluded patients had worse preadmission renal function. This may have caused bias, but these patients did not fulfil the inclusion criterion of expected renal recovery. Because of our small sample size, the multivariate model might be overfitted. However sample size calculation was based on the suggestion of Altman to include a minimum of 70 patients to evaluate 7 variables in multivariate analysis [16]. Furthermore multicollinearity between variables may have affected the results, this was tested and appeared not to be an issue. Unfortunately urinary NGAL concentrations were determined in only 63 patients, because urine production was less than 200 ml/day (which was deemed unreliable) or because of logistic reasons, such as failure to collect or store the urine portion. We cannot exclude that urinary NGAL would be predictive in a larger cohort, but the relation with creatinine clearance and creatinine ratio (day 2/day 0) seems stronger. Furthermore, if urine output is less than 200 ml/day, creatinine clearance is likely low as well. Nevertheless, the group of patients with successful discontinuation was large enough to demonstrate that creatinine clearance or creatinine ratio (day 2/ day 0) and non-renal SOFA score were significant predictors for patients still in the ICU and these relations are clinically plausible. A third limitation is that we do not have data on the day CRRT is discontinued, but on day 2 after discontinuation. The reason that we chose day 2 was that the population still in the ICU is of interest for the intensivist. Our study has several strengths, to our knowledge this is the first prospective multicentre study evaluating predictors of successful discontinuation of CRRT. Second, our study provides a practical tool for the clinician to evaluate whether discontinuation of CRRT will be successful or not, and therefore prevent potentially harmful complications associated with over- or under-treatment of CRRT.

## Conclusions

The present prospective multicentre study found that a calculated 6-h creatinine clearance, a creatinine ratio (day 2/ day 0) and non-renal SOFA score at day 2 after discontinuation independently predicted short-term successful discontinuation of CRRT, while urinary NGAL did not. In our cohort, a creatinine clearance of 11 ml/min (95% CI 6–16 ml/min) and a creatinine ratio (day 2/ day 0) of 1.41 (1.27–1.59) had the optimal cut-off. The study therefore provides a practical bedside tool: discontinuation of CRRT will likely be successful if creatinine clearance is more than 16 ml/min or incremental creatinine ratio (day 2/ day 0) is below 1.27, especially in patients with lower non-renal SOFA score and when fluid balance becomes negative.

## Abbreviations

AKI: Acute kidney injury
APACHE: Acute Physiology And Chronic Health Evaluation
AUC: Area under the curve
CI: Confidence interval
CRRT: Continuous renal replacement therapy
CVVH: Continuous venovenous hemofiltration
CVVHD: Continuous venovenous hemodialysis
eGFR: Estimated glomerular filtration rate
ICU: Intensive care unit
NGAL: Neutrophil gelatinase-associated lipocalin
OR: Odds ratio
ROC: Receiver operator characteristic
SAPS: Simplified Acute Physiology Score
SOFA: Sequential organ failure assessment
VIF: Variance inflation factor
